# Fifty years of research on psychosocial working conditions and health: From promise to practice

**DOI:** 10.5271/sjweh.4180

**Published:** 2024-09-01

**Authors:** Cécile RL Boot, Anthony D LaMontagne, Ida EH Madsen

**Affiliations:** 1 Amsterdam UMC, Department of Public and Occupational Health, Amsterdam, The Netherlands.; 2 Amsterdam Public Health, Societal Participation and Health, Amsterdam, The Netherlands.; 3 Radboud University, Behavioural Science Institute, Work, Health & Performance, Nijmegen, The Netherlands.; 4 Institute for Health Transformation and School of Health & Social Development, Deakin University, Geelong VIC AUSTRALIA.; 5 National Research Centre for the Working Environment, Copenhagen, Denmark.; 6 National Institute of Public Health, University of Southern Denmark, Copenhagen, Denmark.

**Keywords:** psychosocial work environment, psychosocial hazards, mental health, cardiovascular health, intervention, policy

## Abstract

**Objective:**

This paper presents an overview of 50 years of research on psychosocial working conditions and health with regards to conceptualization, interventions and policy. We reflect on the promise of past and current research on psychosocial working conditions and, in addition, discuss current progress in translating this research into workplace practice and improvements in people’s working lives.

**Methods:**

We conducted a narrative review of meta-reviews and key publications on psychosocial working conditions and health. The review covers a historical overview of theories of the past 50 years, measurement of psychosocial working conditions, health effects, intervention research, and policy development on psychosocial working conditions.

**Results:**

Psychosocial working conditions are conceptualized in different ways, with increasing complexity in the understanding developing over time. Exposures related to psychosocial working conditions are associated with a wide range of health outcomes, in particular cardiovascular disease and mental health conditions. In response to growing evidence on associations between psychosocial working conditions and health outcomes, intervention research has expanded rapidly, but for various reasons the evidence base is stronger and more extensive for individual- than organizational-level interventions. This individual/organizational imbalance is reflected in practice, and may partly explain why policy interventions have yet to show reductions in exposures to psychosocial work factors and associated adverse outcomes.

**Conclusions:**

Pressing needs for advancing the field include improvements in capturing exposure dynamics, developing objective measures of exposure, methodologic advancements to optimize causal inference in etiologic studies, and alternatives to randomized controlled trials for intervention evaluation.

Fifty years ago, controlling physical and chemical hazards in the workplace were the main priorities to protect worker health from occupational disease. This is nicely illustrated in the first issue of the *Scandinavian Journal of Work, Environment & Health* (SJWEH), which was filled with studies on exposure to, amongst others, white spirit and asbestos. Shortly before the birth of the journal, the concept of the psychosocial work environment was first articulated in the 1960s (1). Over the past five decades, this area has grown into one of the largest topics in occupational health research, policy and practice. This discussion paper focuses on the psychosocial factors at work, in which we include the way work is designed, organized and managed, as well as the economic and social contexts of work (2).

One of the first SJWEH papers on psychosocial factors at work warned us then of the complexities in this research area due to the challenges related to measuring psychosocial stress, the presence of subjective elements and the large diversity in psychosocial environmental and personal factors at work (3). From this beginning four decades ago, psychosocial factors at work have been a topic of study more often every year (figure 1) and have become the most studied exposure in SJWEH publications over the last decade (4).

For this 50^th^ anniversary of SJWEH, we recount how the research field of psychosocial working conditions has evolved over the past 50 years and where we now stand. We outline the evolution of the conceptualization and measurement of psychosocial working conditions, the identification of associated health outcomes, and development of interventions, policy and practice to prevent and control exposures to adverse psychosocial working conditions and enhance psychosocial working conditions that are beneficial for workers.

**Figure 1:**
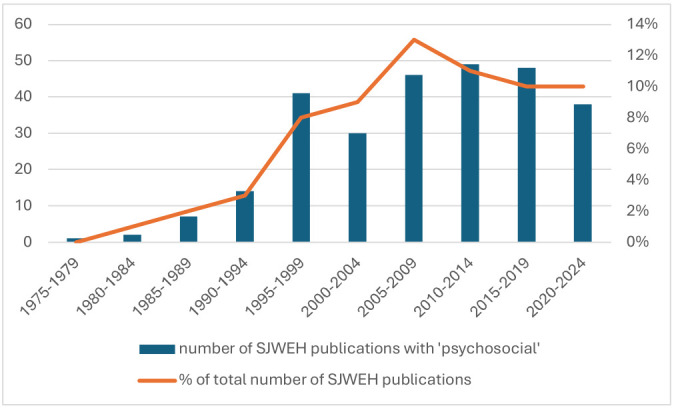
Publications in the *Scandinavian Journal of Work, Environment & Health* retrieved with the search term ‘psychosocial’ as percentage of the total number of papers in the journal from 1975–2024 (N=276, on April 19, 2024).

## Conceptualization and measurement of psychosocial working conditions

**Figure 2:**
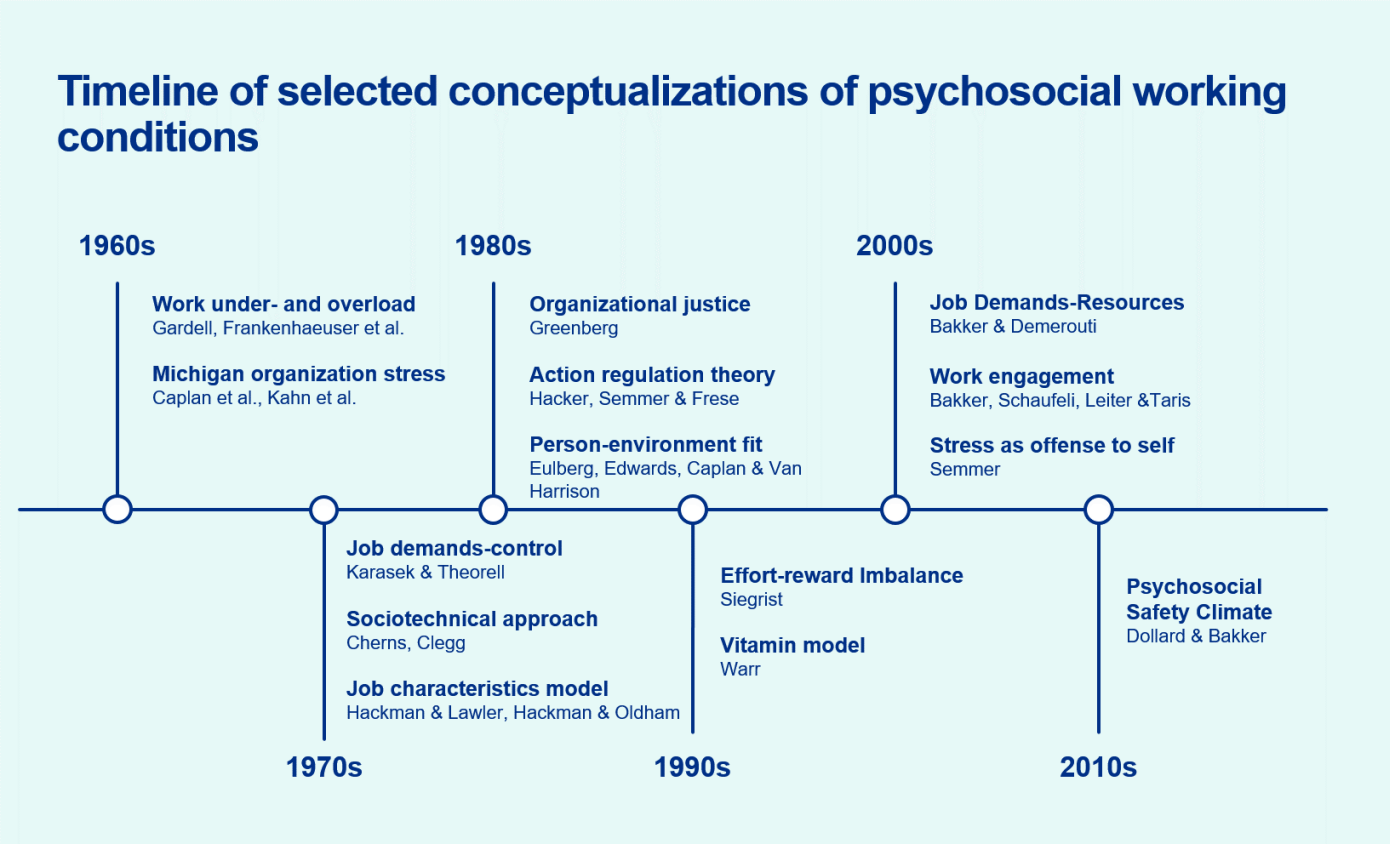
Timeline of selected conceptualizations of psychosocial working conditions. Note: Key references for these conceptualizations are presented in the supplementary material.

Figure 2 depicts a time of selected conceptualizations of psychosocial working conditions. The systematic conceptualization of psychosocial working conditions began in the 1960s, in the work of Gardell & Frankenhaeuser in Scandinavia, for example, concerning work under- and overload, and the work of Kornhauser in the USA (5–7). These concepts laid the foundation for the now seminal model of job strain, which states that work stress may be generated by the combination of high demands and low control at work (8), followed by the inclusion of measures on job security and social support (9, 10). Greenberg added the role of the perception of justice, in the theory of organizational justice (11). According to this theory, it is vital to workers’ wellbeing, performance, and behavior that workers consider their work organization to be just in terms of fairness in how resources are distributed, how procedures and processes are conducted, and how members of the organization are treated (12).

In the mid 1990s, Siegrist added a more sociological perspective: the model of effort–reward imbalance (ERI) (13, 14). This model posits that work stress is produced by breaches of the social contract, in which workers expect a balance between the efforts they put into their work and the rewards they receive in return (13). A later development, the job demands–resources model by Bakker & Demerouti (15), hypothesizes that stress occurs when there is an imbalance between the demands faced by a worker and the resources available to meet those demands. The stress-as-offense-to-self framework (16) is an overarching framework integrating the previously developed models in the field. Developed by Semmer, this framework suggests that work stress arises from a threat to the (social or personal) self-esteem of the person (16). Novel to this framework is the concept of illegitimate work tasks, ie, unreasonable or unnecessary tasks .

The evolution of these models and theories reflects the growing understanding of the various factors and levels shaping the psychosocial work environment. Early models were more task-focused (eg, job strain) with later models aiming to encompass organizational (eg, organizational justice) and labor market levels as well (eg, ERI). To capture the full effects of the psychosocial work environment, more comprehensive approaches have been developed, such as assessing the impacts of combined or multiple exposures as ‘psychosocial job quality’ (17, 18). Useful tools to this end encompass a wide array of psychosocial working conditions, providing for comprehensive assessments of the psychosocial work environment. Examples include the Copenhagen Psychosocial Questionnaire (COPSOQ) (19) (20, 21), the General Nordic Questionnaire for Psychological and Social Factors at Work (QPS-Nordic) (22), and the Danish Psychosocial Work Environment Questionnaire (DPQ) (23). Covering up to 38 dimensions of psychosocial working conditions, these instruments provide validated measures that can be used to examine associations between working conditions and workers’ health and wellbeing. While these instruments are useful for both research and practice, further developments are still needed to establish absolute cut-off points for many of them, as most research is done using categorizations that are sample specific, such as distinguishing between quartiles, tertiles or median split (24).

Perspectives on psychosocial working conditions have also broadened to examine their upstream determinants, ranging from employment conditions such as precarious employment (25), to the influence of the state of the economy and unemployment rates on job insecurity (26–28), to the role of psychosocial working conditions as social determinants of health (29). In summary, much has been gained at the conceptual level in understanding, operationalizing, and measuring the complexity of the psychosocial work environment.

## Identifying consequences of exposure to psychosocial working conditions

Much has also been gained in advancing our knowledge concerning how psychosocial working conditions may affect health over time, and SJWEH has been on the forefront of these developments. In 2004, SJWEH published an early systematic review on the association between psychosocial factors and the risk of cardiovascular disease (30) and, in 2006, the journal published one of the first meta-analyses on the topic (31). Also, Stansfeld & Candy conducted one of the first systematic reviews on psychosocial working conditions and mental health, which SJWEH published in 2006 (32). In 2021, a SJWEH meta-review led by Niedhammer (33) identified 72 systematic reviews concerning psychosocial working conditions and health and documented that reviews had now been conducted in relation to an array of health-related outcomes, including mental conditions (eg, burnout, depression, suicide), health behaviors (eg, smoking, alcohol intake, physical inactivity), various cancers, and cardiovascular disease (eg, stroke, coronary heart disease). The paper concluded that the identified findings were convincing for associations between certain psychosocial working conditions and mental disorders and cardiovascular diseases. For coronary heart disease, a consistently elevated risk was found in relation to job strain and long working hours (33). Also, the meta-review suggested an increased risk of coronary heart disease in relation to job insecurity and organizational justice (33). For stroke, the most consistent finding was an increased risk in relation to long working hours (33).

Rugulies et al's recent umbrella review (34) focused on the relationship between psychosocial working conditions and mental disorders. The paper identified 7 systematic reviews containing 26 pooled estimates of the associations. Overall, increased risk of mental disorder, which was mainly depressive disorder, was found in relation to general psychosocial work stress models such as job strain, ERI, and low procedural justice. Workplace bullying or violence and threats were also associated with increased risk. No association was seen for long working hours.

One key element in advancing the knowledge concerning the relationship between psychosocial work factors and health has been the work of the IPD-Work consortium. Analyzing harmonized data from numerous occupational cohorts, the aim of the consortium was to shed light on the associations between psychosocial work factors and health, using endpoints based on clinical diagnoses. Landmark papers that have become highly cited include the 2012 paper on job strain and coronary heart disease (35), the 2015 paper on long working hours, coronary heart disease and stroke (36), and the 2017 paper on job strain and depression (37). Following their publication, several of the papers were intensively discussed, and there were critiques arguing that the methods applied by the consortium may have led to over- or underestimation of the true associations (38–42). While no methods are completely free from limitations, overall the work of the consortium may be considered to have contributed substantially to the current knowledge base on the association between psychosocial working conditions and clinically significant health endpoints.

While the number of studies reporting associations between psychosocial working conditions and outcomes related to cardiovascular and mental illness is mounting, further research is needed to increase confidence in the causality of the observed associations. Several methodological concerns remain regarding the evidence base. Given the modest magnitude of reported associations, usually with relative risks smaller than 2, residual confounding may affect results. Furthermore, many studies measure working conditions only once, and there is a dearth of studies using repeated measures and examining effects of exposure onset. Finally, most studies measure working conditions using self-reported data, and there is a need for studies using alternative exposure assessment methods to rule out reporting and dependent misclassification (common method variance) biases (34, 43–45).

Despite the typically modest magnitudes of association between psychosocial working conditions and health outcomes, these factors may still be impactful at the population level for common exposures. For example, a recent study from Niedhammer et al (46) estimated that 26% of depression cases in the European working population could be attributable to job strain, ERI, job insecurity, long working hours, and workplace bullying (46). While this estimate rests on the key and controversially discussed (47) assumption that the observed associations are causal, it suggests that there may be considerable preventive potential in reducing exposure to adverse psychosocial working conditions.

Outcomes other than cardiovascular disease and depressive disorder have also been associated with psychosocial working conditions, including musculoskeletal disorders, mortality, and suicide (48–51), which are beyond the scope of this commentary. What is remarkable among exposure to adverse psychosocial working conditions, however, is the number of serious adverse health outcomes that are associated with the same exposures, suggesting that exposure to adverse psychosocial working conditions may be ‘fundamental causes’ of illness in contemporary workplaces (52), and that there could be multiple health benefits of reducing each of these exposures.

## Workplace interventions on improving psychosocial working conditions

The rapidly evolving etiologic evidence base has spurred the growth of intervention research to reduce exposures to psychosocial hazards and their associated adverse impacts on health. As for other occupational hazards, addressing psychosocial working conditions should follow a hierarchy of controls approach. The US National Institute for Occupational Safety and Health, for example, proposed prioritizing elimination, substitution or redesign of the work environment over education of workers and support of adoption of safe and healthy practices (53). In the context of psychosocial work factors, it is important to acknowledge that while preferencing higher level prevention, job demands cannot be eliminated. However, high or excessive job demands can be moderated or mitigated by increasing job control or improving social, emotional or instrumental support. Elimination, substitution or redesign of the workplace or work environment requires interventions at organizational level, whereas education and adoption of healthy practices are mainly directed at the worker. Examples of higher level interventions are reduction of job demands by increasing time allocation for certain tasks or enhancing promotion pathways, whereas interventions targeting lower levels of prevention may include training in anger management, coping abilities, or mindfulness (54).

As suggested by a recent umbrella review (55) and other sources (43, 56–59), the majority of interventions on workplace mental health is on the individual level. The highest quality of evidence was found for interventions targeting individual-level factors rather than organizational-level factors (58, 59). Studying the effects of individual-focused interventions is generally more feasible, and consequently these interventions have more robust options for effectiveness evaluation, resulting in more high-quality studies such as randomized controlled trials. However, effects of individual level interventions on worker health outcomes are often limited, and long-term effects are often small or not measured (56, 60).

A recent umbrella review of interventions at the organizational level focusing on improving psychosocial working conditions over the past two decades reported strong levels of evidence for interventions focusing on changing working time arrangements (55). Moderate quality of evidence was found for interventions focusing on influence on work tasks, work organization or improvements of the psychosocial work environment to enhance worker mental health. Workgroup activities that focused on better communication and support and a participative approach to enhance process aspects in the work environment and core tasks were found to improve the psychosocial work environment. Organizational level intervention entail higher levels of complexity and longer durations. This reduces feasibility of full implementation and limits the possibility to evaluate effectiveness using strong research designs (eg, cluster -randomized controlled trials) (61).

Comprehensive or integrated approaches are needed, in which activities across all levels of an organization are combined to reduce work-related psychosocial hazards (34, 55, 59, 60, 62). The evaluation of such approaches requires the development of robust research designs that can take into account the complexity of such comprehensive approaches without compromising external validity, as well as longer timelines and greater resource requirements for organizational change. Alternatives for randomized controlled trials such as realist evaluation, natural experiments, and target trials may reduce causal inference compared to randomized intervention studies, but the gain in external validity is urgently needed to progress evidence based policy and practice (34, 55, 59–66).

## Impacts on policy level

The accumulating evidence on etiology and interventions has served to justify both regulatory and voluntary policy action around the world. While this is a laudable achievement in itself, pioneers in this field such as Gardell & Levi have sought from the outset for this research to inform evolving policy and practice (43). In this section, we follow this transition and ask what the impacts of this research have been on policy, practice, and people’s working lives.

Sweden was the first country to regulate psychosocial risks at work in the 1970s (67), with other countries following. Most high-income countries have health and safety legislation that applies to workplace psychosocial as well as other risks with more specific standards or regulations in some countries (table 1) (68).

**Table 1:** Selected examples of national standards and regulatory interventions on psychosocial work factors.

Country	Year	Description	Reference
Sweden	1974	Emphasized organizational aspects of psychosocial risk, including focus on managers to prevent and take action against psychosocial risks, with social partner collaboration. Refinements in 1977, 1993 and 2015.	(67, 90)
Belgium	1997	Preventive approach to psychosocial risks, mandating risk assessment and management with the involvement of workers and their trade union representatives. Expressed the complementary roles of primary, secondary and tertiary prevention. Acknowledged the multiple forms of psychosocial risk.	(67)
UK	1999	“Health & Safety at Work” regulations and “Management Standards” require employers to assess the risk of stress-related ill health arising from work	(91)
Japan	2015	“Stress Check Program” implemented to monitor and prevent workplace psychosocial stress at workplaces	(92)
Denmark	2020	“Executive order on psychosocial working environment”, with particular emphasis on: Heavy workload and time pressure; Unclear and conflicting demands at work; High emotional demands when working with people; Offensive behaviour, including bullying and sexual harassment; Work-related violence	(93)
Australia	2022	National “Code of Practice for Managing Psychosocial Hazards at Work” provides guidance on psychosocial risk assessment and management, but is only mandated if enacted into legislation at state or territory level.	(94)

Parallel to regulation, various voluntary or ‘soft’ policies have been put forth. The European Framework for Psychosocial Risk Management (PRIMA-EF) was a valuable example of research translation to policy (69). PRIMA-EF was developed over 2006–2009 by an international consortium of researchers, social partners, and other stakeholders including the World Health Organization (WHO) and the International Labor Organization (ILO). Best practice guidance and other materials were widely disseminated to European workplaces.

In 2013, Canada’s Mental Health Commission issued its *National Standard for Psychological Health & Safety in the Workplace* (70), which outlined an approach to develop and sustain psychologically healthy and safe workplaces, focusing not only on psychosocial working conditions but also on mental illness prevention and mental health promotion. Australia’s 2021 *National Workplace Initiative* was also driven by a national Mental Health Commission, similarly including workplace psychosocial risk management but with an overarching aim “to provide a nationally consistent approach to workplace mental health” (71).

While attention from mental health authorities is welcome and can powerfully complement occupational health and safety policy, this also represents a subtle shift in emphasis from focusing on psychosocial working conditions to focusing on mental health and illness, with a concomitant shift in emphasis from work-directed to individual- and illness-directed interventions. This shift risks detracting from the primary legal and ethical obligation of employers: to provide working conditions that are both physically and psychologically safe. An exclusive mental health focus also ignores the impacts of psychosocial working conditions on cardiovascular disease, mortality and other adverse outcomes. Contributing reasons for this shift likely include the growing societal recognition of the widespread prevalence and impacts of mental disorders, and the rapid growth of stress-related workers’ compensation claims for adverse mental health conditions and associated costs in high-income countries (34). Further, this shift has likely been enhanced by the relative strengths of the individual-directed evidence base compared to organizational-level evidence base, as described above.

Other recent voluntary policy initiatives include the International Standards Organisation’s (ISO) standard for managing psychosocial risk at work, which has a strong focus on psychosocial working conditions and acknowledges the full range of adverse health and organizational impacts (72). The 2022 WHO *Guidelines on Mental Health at Work* (73) again focuses on mental health and illness only, but it is complemented by a stronger emphasis on psychosocial working conditions in a companion joint WHO/ILO policy brief (74).

To what extent have these various policy interventions shifted practice? There has been relatively little population-level implementation evaluation, and – where it exists – it tends to be in the grey literature. For example, a survey of 1899 UK private businesses found that 31% had heard of the Management Standards (table 1), and only 7% had used them (75). In general, available evidence suggests that common practice is disproportionately individual- and illness-directed, with less organizational-level intervention targeting the reduction of exposures to psychosocial working conditions (56, 58, 62). This does not align with best practice, which recommends a comprehensive or integrated work-, worker-, and illness-directed intervention (34, 60, 76). This represents both a practice and a research gap, which warrants increased research, policy and practice attention.

To what extent have policy interventions been associated with improvements in psychosocial working conditions? Again, there has been relatively little research on this to date (77). Most of the available time trend/surveillance studies shows either stable (eg, in Australia over 2001–2008) (28) or deteriorating psychosocial working conditions—particularly for lower status workers (eg, in a European study over 2005–2010) (78), with relatively little assessment of these trends in relation to policy intervention. Other studies suggest that psychosocial working conditions are largely deteriorating in Spain, the USA and Canada and that inequalities in conditions may be widening in the USA and Europe (77). A review of a number of studies relating psychosocial working conditions to country-level investment in active and passive labor market programs found that low investment countries saw deteriorations whereas high investment countries remained stable (79). In Sweden, arguably one of the policy leaders internationally, a 2021 study suggested that exposures to psychosocial working conditions were generally stable over the period from 1997–2015, and that there was little evidence of widening inequalities (80). In light of the likely drivers of deteriorating conditions globally, including the rise of neoliberalism, deregulation and globalization over recent decades (29, 81–83), it could be that Sweden and other countries warding off such declines represents a success.

Though there are few studies focused explicitly on regulatory policy interventions (84), signs are promising. A key informant study found that greater national policy attention was associated with better enterprise-level psychosocial safety climate (85). Policy attention, however, was found to be mainly focused on physical violence, discrimination, harassment and bullying at work, which tend to be event-based occurrences. More chronic psychosocial working conditions such as job control tended to receive far less policy attention. The same study (85) also replicated an earlier finding that union density was associated with better psychosocial safety climate (86), suggesting complementary roles for socio-political and policy attention to improve the psychosocial safety climate.

Finally, a study across 35 European countries integrated implementation and effectiveness questions (87), finding that, in countries with specific regulatory policies on psychosocial risk or work-related stress, enterprises were more likely to have action plans to reduce work-related stress, as well as being more likely to have better psychosocial working conditions and less reported work-related stress among at the worker level. It was also observed that interventions tended to be more individual- than organizational-directed. This recurring theme of an imbalance between individual and organizational interventions (referred to above), suggests that further preventive potential could be realized.

In summary, the available evidence indicates that the vast etiologic and intervention research evidence base has not substantially translated to reduced psychosocial risk in the workplace. Though there are promising signs where policy attention has been the greatest, there is far less evidence—as well as less policy attention—in other parts of the world. Increased research on policy development, implementation and evaluation, and the role of associated social and political conditions, could aid the translation of research to practice. This would include monitoring and surveillance (77, 88), policy evaluation (89), and investigation of innovative strategies to support best practice (34).

## Concluding remarks

Serendipitously, the birth of SJWEH coincided with the birth of psychosocial working conditions as a research field. In 50 years, we have gone from little understanding to a range of ways to conceptualize and measure psychosocial working conditions. We have learnt that these exposures are associated with a wide range of health outcomes, in particular cardiovascular disease and mental health conditions. In response, intervention research has expanded rapidly, but – for various reasons – the evidence base is stronger and more extensive for individual- than organizational-level interventions. This individual/organizational imbalance is reflected in practice and may partly explain why policy interventions have yet to show reductions in exposures to psychosocial work factors and associated adverse outcomes. Pressing needs for advancing the field are presented in the box below. We look forward to seeing the promise of this concerted research effort manifesting in workplace practice and improvements in people’s working lives.

**Future research needs on psychosocial working conditions**

**Conceptualization and measurement:**
- Better capture exposure dynamics using repeated measures;
- Assess exposures by means other than self-report;
- Develop combined measures of multiple exposures (co-exposures, clustering, etc).

**Identifying consequences of exposure to psychosocial working conditions:**
- Advance analytics to optimize causal inference (eg, target trials);
- Account comprehensively for confounding by non-work factors.

**Interventions, policy and practice:**
- Target multiple levels such as organizations, business units, and at the worker level;
- Target mitigation of excessive job demands;
- Apply participatory/co-design approaches including stakeholders;
- Develop alternatives to experimental studies for intervention evaluation (eg, realist evaluation);
- Exploit natural experiments to evaluate policy and other interventions.

## Supplementary material

Supplementary material
