# Supplementary material for: Fifty years of research on psychosocial working conditions and health: From promise to practice
Source: Scand J Work Environ Health. 2024 Aug 30;50(6):395–405. doi: 10.5271/sjweh.4180 (PMC11389251; doi:10.5271/sjweh.4180)
Supplement: Supplementary material [file SJWEH-50-395-S001.pdf]

Fifty years of research on psychosocial working conditions and health: From promise to practice[1]

by Cécile RL Boot, PhD,[2] Anthony D LaMontagne, PhD, Ida EH Madsen, PhD

1. Key references in relation to figure 2
2. Correspondence to: Cécile RL Boot, Amsterdam UMC, Department of Public and Occupational Health, Amsterdam, The Netherlands. [E-mail: crl.boot@amsterdamumc.nl]

Concept	References
Work under- and overload	Frankenhaeuser M, Gardell B (1976). Underload and overload in working life: outline of a multidisciplinary approach. Journal of Human Stress 2(3):35-46.
Michigan organization stress	Caplan, R., Cobb, S., French, J. & Harrison, R. (1975) Job Demands and Worker Health, Main Effects and Occupational Differences. Washington, DC: NIOSH.<br><br>Kahn, R.L., Wolfe, D., Quinn, R., Snoek, J. & Rosenthal, R. (1964) Organizational Stress: Studies in Role Conflict and Ambiguity, New York: John Wiley & Sons.
Job demands-control	Karasek RA. Job Demands, Job Decision Latitude, and Mental Strain: Implications for Job Redesign. Administrative Science Quarterly. 1979;24(2):285-308.<br><br>Karasek R, Theorell T: Healthy work: stress, productivity, and the reconstruction of working life. New York, Basic Books; 1990.
Sociotechnical approach	Cherns, A.B. (1976) The principles of sociotechnical design. Human Relations 29:783–92.<br><br>Cherns, A.B. (1987) Principles of sociotechnical design revisited. Human Relations 40:153–62.<br><br>Clegg, C.W. (2000) Sociotechnical principles for system design. Applied Ergonomics 31:463–77.
Job characteristics model	Hackman, J.R. & Lawler, E.E. (1971) Employee reactions to job characteristics. Journal of Applied Psychology 55:259–86.<br><br>Hackman, J.R. & Oldham, G.R. (1975) Development of the Job Diagnostic Survey. Journal of Applied Psychology 60:159–70.<br><br>Hackman, J.R. & Oldham, G.R. (1976) Motivation through the design of work: test of a theory. Organizational Behavior and Human Performance 16:250–79.<br><br>Hackman, J.R. & Oldham, G.R. (1980) Work Redesign. Reading, MA: Addison-Wesley.
Organizational justice	Greenberg J. A. (1987) Taxonomy of Organizational Justice Theories. The Academy of Management Review 12(1):9-22.

	Elovainio M, Kivimäki M, Vahtera J. (2002) Organizational justice: evidence of a new psychosocial predictor of health. American Journal of Public Health 92(1):105-8.
Action-theoretical approach	Hacker, W. (1985) Activity: a fruitful concept in industrial psychology. In M. Frese & J. Sabini (eds) Goal Directed Behavior: A Concept of Action Psychology. Hillsdale, NJ: Lawrence Erlbaum, pp. 262–83.<br><br>Semmer, N. & Frese, M. (1985) Action theory in clinical psychology. In M. Frese & J. Sabini (eds) Goal Directed Behavior: The Concept of Action in Psychology. Hillsdale, NJ: Lawrence Erlbaum, pp. 296–310.
Person & environment fit	Eulberg J.R., Weekley J.A., Bhagat R.S. (1988) Models of Stress in Organizational Research: A Metatheoretical Perspective. Human Relations 41(4):331-50.<br><br>Edwards J.R., Caplan R.D., Harrison R.V. (1998) Person-environment fit theory: Conceptual foundations, empirical evidence, and directions for future research. Theories of organizational stress 28:28-67.
Effort-reward imbalance	Siegrist J. (1996) Adverse health effects of high-effort/low-reward conditions. Journal of Occupational Health Psychology 1(1):27-41.
Vitamin model	Warr, P. (1987) Work, Unemployment and Mental Health. Oxford: Clarendon Press.<br><br>Warr, P. (1994) A conceptual framework for the study of work and mental health. Work and Stress 8:84–97.<br><br>Warr, P. (1996) Employee well-being. In P. Warr (ed.) *Psychology at Work*, 4th ed. Chichester: John Wiley & Sons.
Psychological safety	Edmondson A. (1999) Psychological safety and learning behavior in work teams. Administrative science quarterly 44(2):350-83.
Job demands & resources	Bakker A.B., Demerouti E. (2007) The job demands-resources model: State of the art. Journal of Managerial Psychology 22(3):309-28.
Work engagement	Bakker A.B., Schaufeli W.B., Leiter M.P., Taris T.W. (2008) Work engagement: An emerging concept in occupational health psychology. Work & Stress 22(3):187-200.
Stress as offense to self	Semmer N., Jacobshagen N., Meier L., Elfering A. (2007) Occupational stress research: The "Stress-as-Offense-to-Self" perspective. In: McIntyre, S.; Houdmont, J. (eds.) Occupational Health Psychology: European Perspectives on Research, Education and Practice (Vol. 2) (pp. 41-58). Nottingham: Nottingham University Press.
Psychosocial safety climate	Dollard MF, McTernan W. (2011) Psychosocial safety climate: a multilevel theory of work stress in the health and community service sector. Epidemiology and Psychiatric Sciences 20(4):287-93.
